# JMJD3 and NF-κB-dependent activation of Notch1 gene is required for keratinocyte migration during skin wound healing

**DOI:** 10.1038/s41598-017-06750-7

**Published:** 2017-07-26

**Authors:** Jungtae Na, Jee Yoon Shin, Hayan Jeong, Jee Youn Lee, Beom Joon Kim, Won Sun Kim, Tae Young Yune, Bong-Gun Ju

**Affiliations:** 1 Department of Life Science, Sogang University, Seoul, 04107 Korea; 2 Age-Related and Brain Diseases Research Center, School of Medicine, Kyung Hee University, Seoul, 02447 Korea; 3 Department of Dermatology, College of Medicine, Chung-Ang University, Seoul, 06973 Korea

## Abstract

It has been shown that epigenetic regulation plays an important role in skin wound healing. We previously found that histone H3K27me3 demethylase JMJD3 regulates inflammation and cell migration in keratinocyte wound healing. In this study, we identified Notch1 as a direct target of JMJD3 and NF-κB in wounded keratinocytes using *in vitro* cell and *in vivo* animal models. We found that Notch1 is up-regulated in the wound edge and its expression is dependent on JMJD3 and NF-κB in wounded keratinocytes. We also found that Notch1 activates the expression of RhoU and PLAU gene, which are critical regulators of cell migration. Consistently, depletion or inactivation of Notch1 resulted in decreased filopodia formation, increased focal adhesion and actin stress fiber, leading to reduced keratinocyte migration and skin wound healing. Thus, our findings provide the molecular mechanism involving JMJD3/NF-κB-Notch pathway in keratinocyte wound healing.

## Introduction

The skin forms physical and immunological barriers from the external environment to maintain water balance, body temperature, insulation, sensation, and immunity^1, 2^. When the skin barrier is disturbed, wound healing begins immediately after injury. The skin wound healing process occurs in four overlapping stages: haemostasis, inflammation, migration and proliferation, and remodeling^3, 4^. Within an hour after injury, blood coagulation and platelet activation at the injury site form a fibrin clot, which stops the bleeding and fills the discontinuity in the skin. The activated platelets provide various growth factors required for inflammation, angiogenesis, and migration of keratinocytes and fibroblasts^5^. During the inflammation stage, bacteria and cell debris are cleaned by activated neutrophils from injured blood vessels. In the migration and proliferation stage that follows, keratinocytes at the wound edge migrate into the wound via the changing of cell-cell and cell-matrix interaction through up-regulation of matrix metalloproteinases (MMPs). In addition, keratinocytes distant from the wound edge actively proliferate, resulting in the filling of the wound gap. Accumulating evidence indicates that stem cells from the bulge of hair follicles contribute to re-epithelialization^6, 7^. Once the wound gap is filled, the basement membrane is re-established, and keratinocytes are differentiated. Concurrently, tissue granulation and angiogenesis take place. During wound contraction, myofibroblasts help to decrease the size of the wound. In the maturation and remodeling stage, the replacement of collagen and rearrangement of collagen fibers occur, forming a tension line that increases the tensile strength of the wound.

Accumulation evidences have revealed that Notch signaling plays an important role in skin development, homeostasis, and wound healing by the regulation of differentiation, proliferation, and apoptosis^8–11^. Mammals have four Notch receptors (Notch1~4) and five ligands such as Delta-like 1, 3, and 4, and Jagged 1 and 2. To activate Notch signaling, the interaction between a transmembrane Notch receptor and a transmembrane ligand is required on two adjacent cells. Ligand binding to Notch receptor results in successive proteolytic cleavage of Notch at extracellular and transmembrane domain, producing Notch intracellular domain (NICD). Translocated NICD to the nucleus form a complex with RBP-J transcription factor and regulates target gene expression including HES and Hey family. In fact, Notch family genes and their ligands are expressed in various epidermal layers during development^12–16^. Consistently, abnormal Notch signaling results in impaired epidermal differentiation, proliferation, and inflammation, leading to a variety of skin diseases including skin cancer, psoriasis, and atopic dermatitis^10, 11, 17^.

Recently, epigenetic regulation, such as DNA methylation and histone modification, has been investigated in skin wound healing^18, 19^. DNA methylation mediated by DNA methyltransferases (DNMTs) has an important role in the maintenance of epidermal progenitor self-renewal during embryonic development^20^. However, very few reports demonstrated that DNA methylation is implicated on skin wound healing. For example, decreased level of global DNA methylation is observed in rat skin wound healing^21^. In contrast to DNA methylation, several reports demonstrated the importance of histone modification or histone-modifying enzymes in skin wound healing. Reduction of histone H3K27 trimethylation is observed in the murine skin wound epidermis^22^. Consistently, Polycomb group protein (PcG) such as Eed, Ezh2, and Suz12 are down-regulated while H3K27 demethylases such as JMJD3 and UTX are up-regulated, resulting in Myc and Egfr gene activation^22^. Up-regulated JMJD3 at wound edge is required for the inflammatory, MMP, and growth factor gene activation in keratinocytes after wounding^23^. In addition, double knock out mice for Ezh1 and Ezh2 histone methyltransferase clearly show defective cell proliferation and wound healing^6^. Regulation of histone acetylation also is shown to be crucial for skin wound healing. Expression of HDAC2 (histone deacetylase 2) increases at the wound margin in early phase and in the wound bed at later stages^24^. Interestingly, class I-IIa HDAC inhibition by trichostatin A (TSA) results in accelerated wound healing through displacement of HDAC2 from gene promoters and up-regulation of IGF-I, FGF-10, and EGF gene expression^25^. However, this effect is suppressed by class III HDAC (SIRTs) inhibitor, indicating that SIRT may be an upstream negative regulator of class I HDAC in wound healing^25^. The level of histone H3K9 acetylation is increased in the wound area and the application of p300/PCAF activator on wounded skin induces accelerated wound healing^26^. In contrast, the deletion of Setd8 histone methyltransferase, which is essential for epidermal progenitor cells, is not altered in the wound healing process^27^.

In this study, we demonstrated that JMJD3 and NF-κB-dependent Notch1 activation is required for the regulation of focal adhesion, filopodia, and stress fiber formation though RhoU and uPA gene expressions, leading to keratinocyte migration during the early phase of skin wound healing.

## Results

### Up-regulation of Notch expression in wounded keratinocytes

**Figure 1:**
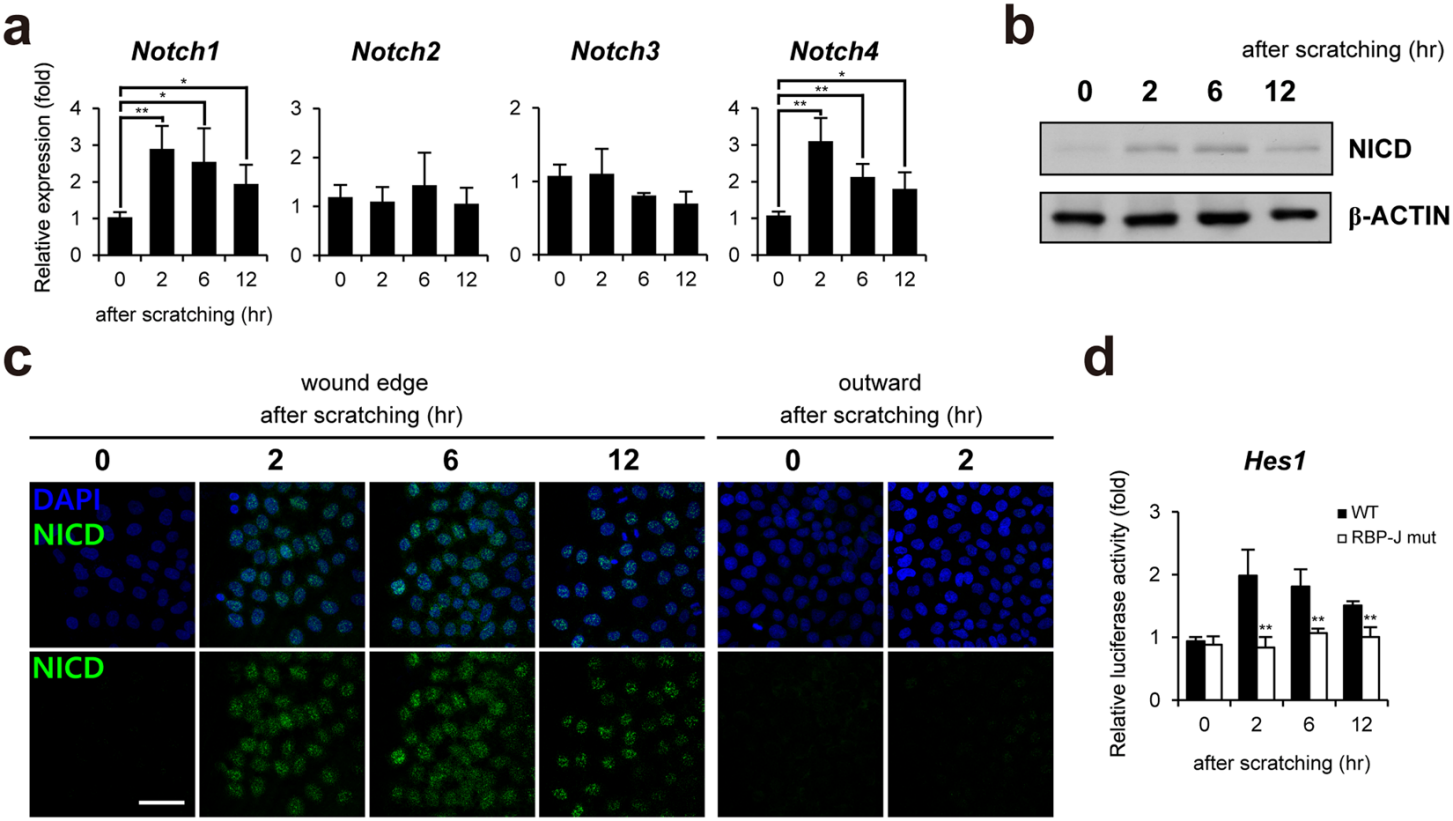
Up-regulation of Notch1 expression in scratch-wounded keratinocytes. (**a**) After HaCaT keratinocytes were scratched, transcripts of *Notch1*~*4* and *GAPDH* were determined by quantitative PCR. (**b**) Lysates from scratch-wounded HaCaT keratinocytes were immunoblotted with anti-NICD and anti-β-ACTIN antibodies, respectively. (**c**) Increased nuclear NICD expression in the region of the wound edge compared to the region far from the wound edge (outward) (>1 mm). HaCaT keratinocytes after scratching were immunostained with anti-NICD antibody. Nuclei were identified using DAPI staining. Scale bar, 25 μm. (**d**) Up-regulation of *Hes1* promoter reporter activity after scratching. However, activity of promoter reporter containing mutated RBP-J binding sites is not up-regulated by scratching.

Although Notch signaling plays an important role in epidermal differentiation and skin maintenance, the molecular mechanism underlying Notch-mediated keratinocyte wound healing has not been fully elucidated. Thus, we first examined the expression of *Notch* genes in the scratch-wounded HaCaT keratinocytes. Quantitative PCR analysis revealed that the expression of *Notch1* and *Notch4* genes increased acutely at 2~6 hr after scratching and then slowly decreased (Fig. 1a). Since *Notch1* is expressed more abundantly than *Notch4* (Supplementary Fig. S1) and a primary Notch receptor in epidermal differentiation, proliferation, and inflammation^12, 15, 28–30^, we further examined expression of NICD (Notch intracellular domain) of Notch1 at protein level. Similarly, Western blot analysis showed the acute up-regulation of NICD expression (Fig. 1b). We also observed increased nuclear expression of NICD in the region of the leading wound edge, while no elevated expression were detected in the region far from the wound edge as well as in confluent monolayer HaCaT keratinocytes (Figs 1c and S2). In addition, the promoter activity of *Hes1* gene, a direct target of Notch, was up-regulated acutely at 2~6 hr after scratching (Fig. 1d). Mutation of RBP-J binding sites on the *Hes1* gene promoter abolished the scratch-induced activation of the promoter activity.

### JMJD3 activates expression of Notch1 gene in wounded keratinocytes

**Figure 2:**
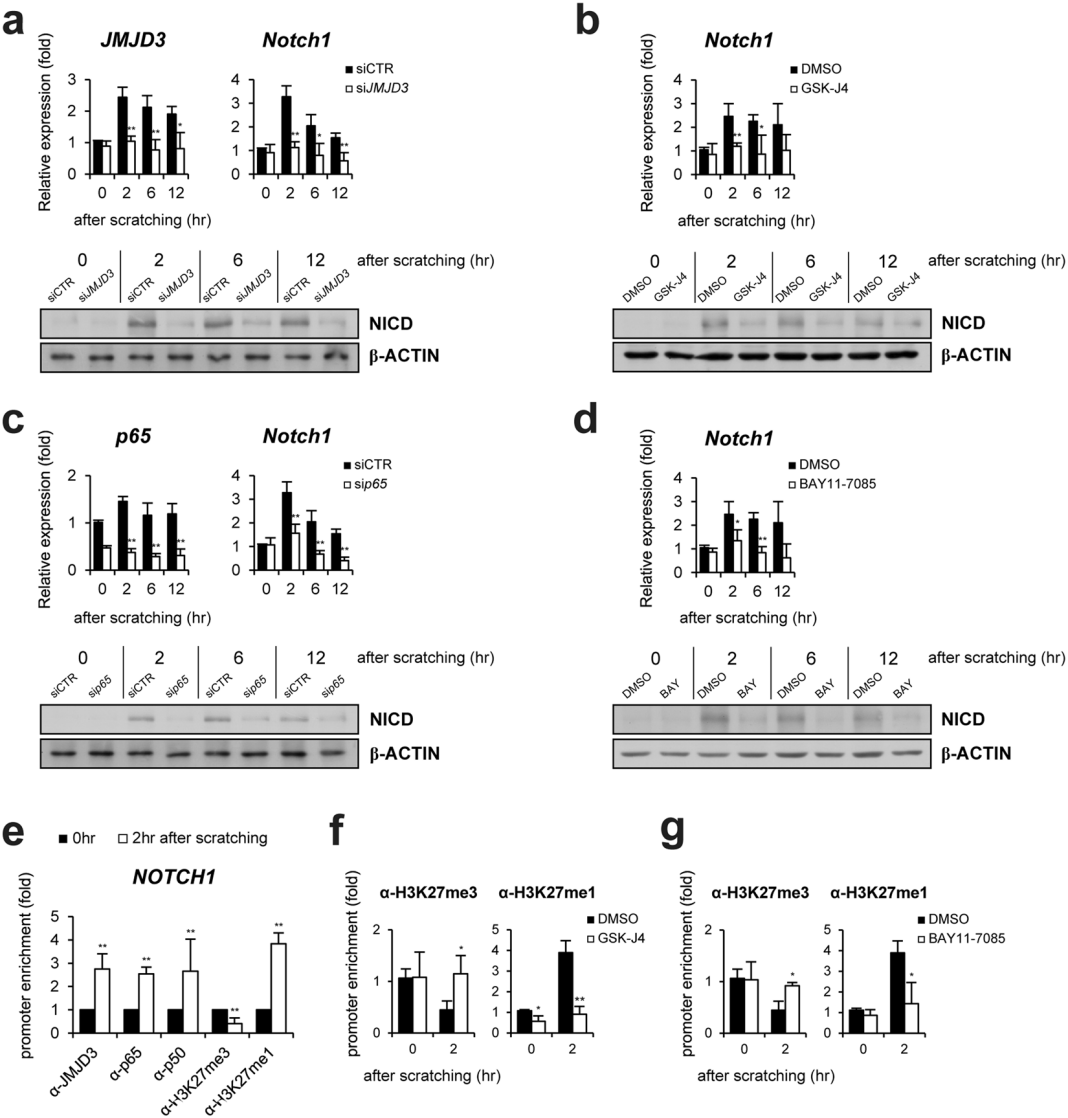
JMJD3 and NF-κB are required for up-regulation of Notch1 expression in scratch-wounded keratinocytes. (**a**,**b**) Following the depletion (siJMJD3) or inactivation (GSK-J4) of JMJD3, HaCaT keratinocytes were scratched and expression level was determined by quantitative PCR and Western blotting. (**c**,**d**) Following depletion (sip65) or inactivation (BAY11-7085) of NF-κB, HaCaT keratinocytes were scratched and expression level was determined by quantitative PCR and Western blotting. (**e**) ChIP assay was performed in scratch-wounded HaCaT keratinocytes. The occupancy of each protein was determined with quantitative PCR at the *Notch1* gene encompassing the NF-κB binding sites. ChIP using normal IgG was performed as a negative control. (**f**,**g**) The methylation level of H3K27 was measured by ChIP assay in scratch-wounded HaCaT keratinocytes treated with GSK-J4 or BAY11-7085.

Since the spatiotemporal expression pattern of Notch1 is similar with that of JMJD3 in the early phase of wounded HaCaT keratinocytes^23^, we next tested whether JMJD3 regulates the *Notch1* gene expression. When the transcript of JMJD3 was depleted by RNA interference, the expression of *Notch1* decreased in the scratch-wounded keratinocytes at RNA and protein level (Fig. 2a). Decreased expression of nuclear NICD was observed in the region of the leading wound edge (Supplementary Fig. S3a). We also utilized GSK-J4 to inhibit the histone demethylase activity of JMJD3^31^. GSK-J4 treatment reduced *Notch1* expression (Figs 2b and S3b). JMJD3 has been shown to interact with NF-κB and up-regulate gene expression of MMP, inflammatory, and growth factor during keratinocytes wound healing^23^. Thus, we also tested the dependency of NF-κB for the *Notch1* gene activation. Depletion of NF-κB p65 suppressed *Notch1* gene activation and nuclear expression of NICD in wounded keratinocytes (Figs 2c and S3c). Consistently, BAY11–7085, which inhibits IκBα phosphorylation, showed same effects of NF-κB p65 depletion (Figs 2d and S3d). We also found suppression of promoter activity of *Notch1* gene by depletion or inactivation of NF-κB p65 (Supplementary Fig. S4).

To investigate whether JMJD3 and NF-κB directly activates *Notch1* gene expression, we identified NF-κB binding sites on the upstream region of *Notch1* gene (Supplementary Fig. S7a)^32^. The recruitment of JMJD3 and NF-κB p65 to the upstream region of *Notch1* gene was investigated in HaCaT keratinocytes after scratching by a ChIP assay. Chromatin from HaCaT keratinocytes at 0 and 2 hr after scratching were immunoprecipitated. Quantitative PCR showed that JMJD3, p50, and p65 are significantly bound to the NF-κB binding sites of the *Notch1* gene (Fig. 2e). Consistent with the recruitment of JMJD3, we detected a decreased level of tri-methylated H3K27 (H3K27me3) as well as an increased level of mono-methylated H3K27 (H3K27me1) at the promoter (Fig. 2e). Inactivation of JMJD3 or NF-κB consistently showed the maintenance of the tri-methylation level of H3K27 in the upstream region of *Notch1* gene (Fig. 2f,g).

### Notch1 regulates keratinocyte wound healing via cell migration

**Figure 3:**
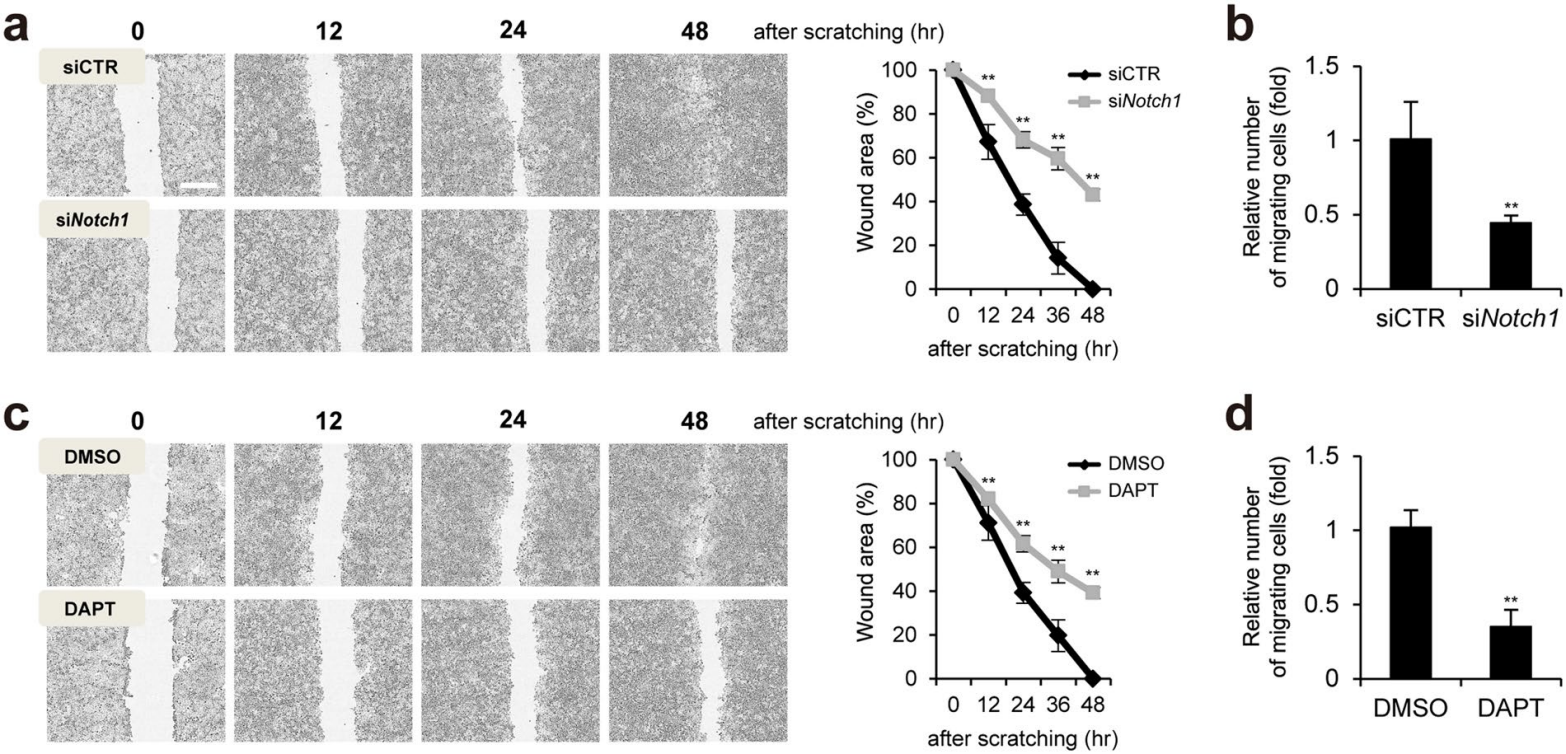
Notch1 regulates keratinocyte migration in scratch-wounded keratinocytes. (**a**,**b**) Depletion of Notch1 (siNotch1) attenuates HaCaT keratinocyte migration after scratching. HaCaT keratinocytes were scratched and photographed. Scale bar, 400 μm. Transwell assay was performed. (**c**,**d**) Inactivation of Notch1 (DAPT) attenuates HaCaT keratinocyte migration after scratching. Transwell assay was performed.

We investigated the effect of Notch1 depletion or inactivation on the keratinocytes migration in response to scratching. As shown in Fig. 3, wound closure was significantly delayed in Notch1-depleted HaCaT keratinocytes as compared to control siRNA-transfected keratinocytes (Fig. 3a). Transwell migration assay also showed significantly fewer migrating keratinocytes in Notch1-depleted keratinocytes (Fig. 3b). Inhibition of Notch signaling by DAPT, a γ-secretase inhibitor, resulted in delayed wound closure and suppressed keratinocyte migration (Fig. 3c,d). In contrast, over-expression of NICD in HaCaT keratinocytes accelerated wound closure as compare to control empty vector-transfected keratinocytes (Supplementary Fig. S5).

### Notch regulates keratinocytes migration via formation of filopodia, focal adhesion, and actin stress fibers

**Figure 4:**
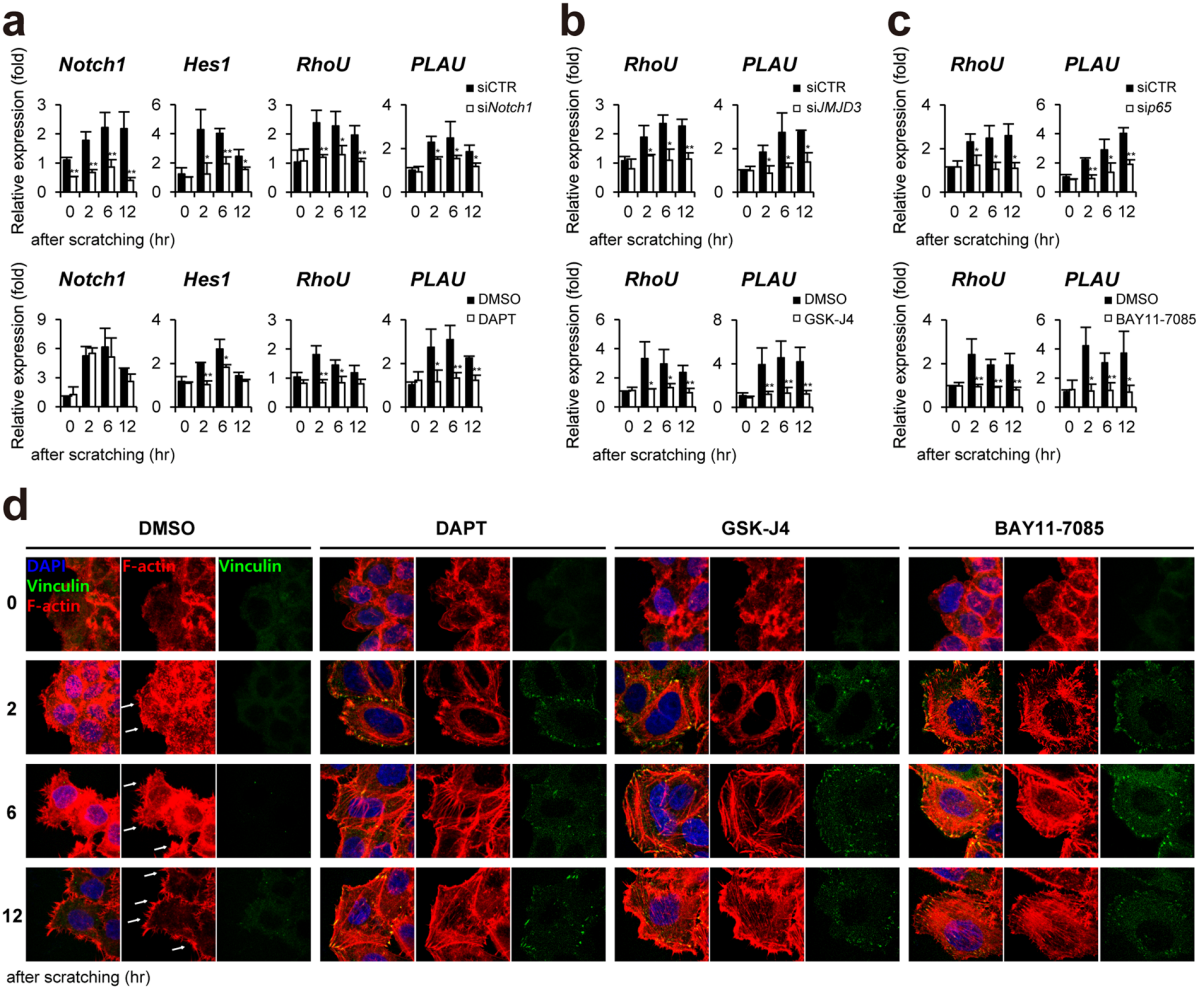
Notch1 regulates keratinocytes migration via RhoU and PLAU gene expression in scratch-wounded keratinocytes. (**a**) Depletion (siNotch1) or inactivation (DAPT) of Notch1 (**b**) Depletion (siJMJD3) or inactivation (GSK-J4) of JMJD3 (**c**) Depletion (sip65) or inactivation (BAY 11-7082) of NF-κB suppresses *RhoU* and *PLAU* gene activation in scratch-wounded HaCaT keratinocytes. Transcripts of *Notch1*, *Hes1*, *RhoU*, *PLAU*, and *GAPDH* were determined by quantitative PCR. (**d**) Inactivation of Notch1 or JMJD3 or NF-κB results in decreased filopodia-like protrusions (white arrows), increased focal adhesion and actin stress fibers in scratch-wounded HaCaT keratinocytes. To detect filopodia and actin stress fibers, cells were stained with phalloidin (red). Focal adhesion was identified by cell immunostaining with anti-vinculin antibody (green). Nuclei were identified using DAPI staining. Scale bar, 25 μm.

We first conducted a literature search to identify Notch1 target genes, which affect directly cell migration^33–39^. Among them, we found that expression of *RhoU* Rho GTPase (also known as Wrch1) and *PLAU* (plasminogen activator, urokinase) genes are dependent on Notch1 in scratch-wounded HaCaT keratinocytes (Fig. 4a). Consistently, JMJD3 depletion or inhibition suppressed *RhoU* and *PLAU* gene activation (Fig. 4b). NF-κB p65 depletion or inhibition showed the same effect on the *RhoU* and *PLAU* gene expression (Fig. 4c).

RhoU has been shown to regulate cell migration through formation of filopodia, focal adhesion, and dissolution of stress fiber in diverse cell types^40–42^. In addition, PLAU converts plasminogen to plasmin, which regulates cell adhesion and migration though cleavage of ECM and MMPs^43^. Therefore, we investigated whether Notch regulates formation of filopodia, focal adhesion, and actin stress fibers in the scratch-wounded HaCaT keratinocytes using cell staining with anti-vinculin antibody and phalloidin. As shown in Fig. 4d, we observed that control HaCaT keratinocytes show well developed filopodia-like protrusion, which is stained by phalloidin, at the leading edge. However, fewer filopodia were observed in DAPT, GSK-J4, and BAY11–7085-treated keratinocytes, respectively (Fig. 4d). In addition, DAPT, GSK-J4, and BAY11–7085 treatment significantly increased the number of focal adhesion stained with anti-vinculin antibody (Fig. 4d). Similarly, increased bundles of stress actin filaments were observed in DAPT, GSK-J4, and BAY11-7085-treated keratinocytes compared with control HaCaT keratinocytes after scratching (Fig. 4d). We found consistently that depletion of RhoU or PLAU results in delayed wound closure as compared to control siRNA-transfected HaCaT keratinocytes (Supplementary Fig. S6).

### Recruitment of NICD to the RhoU and PLAU gene promoters in wounded keratinocytes

**Figure 5:**
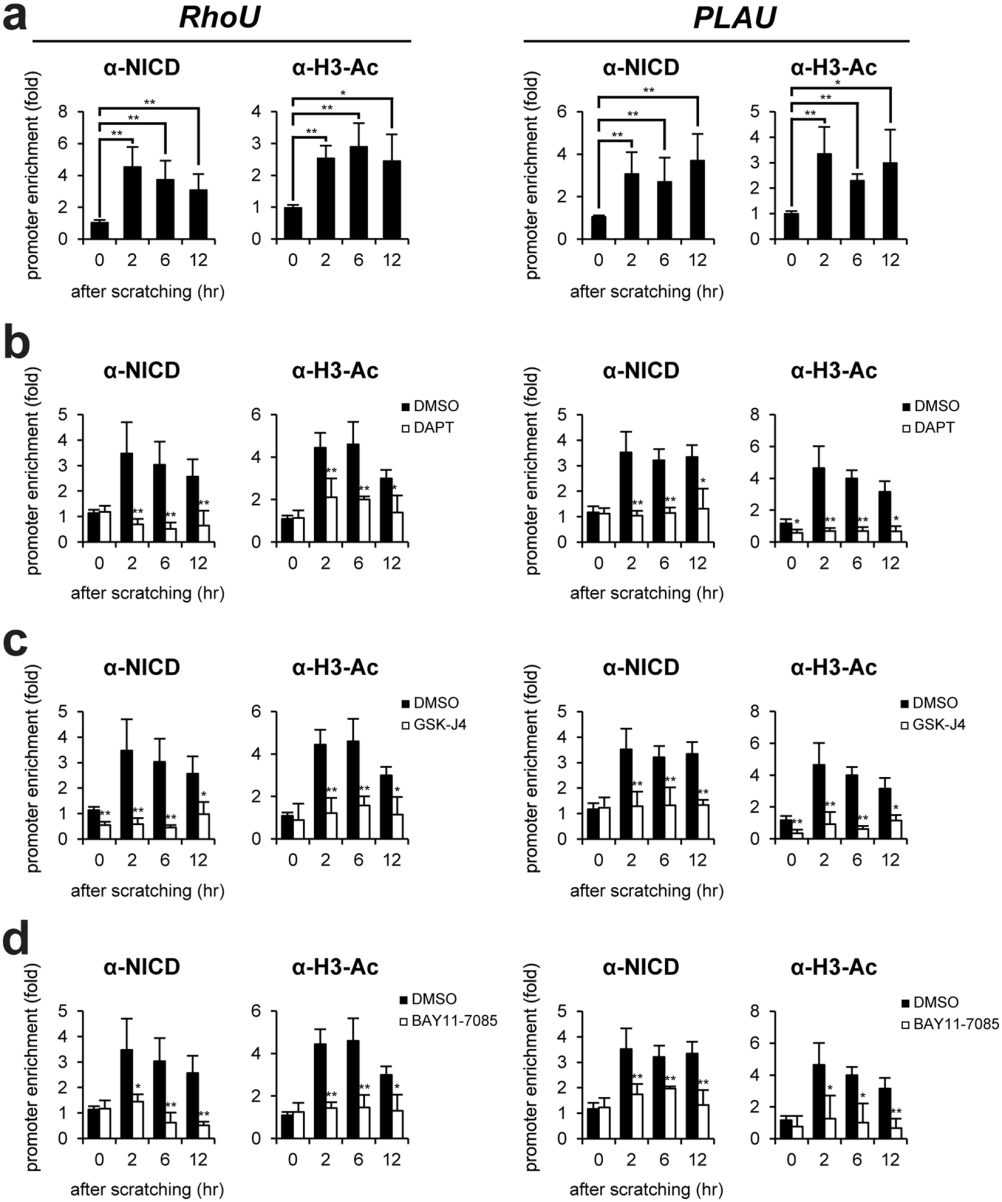
Notch1 activates RhoU and PLAU gene expression in scratch-wounded keratinocytes. (**a**) ChIP assay was performed in scratch-wounded HaCaT keratinocytes using the anti-NICD and anti-acetylated H3 antibodies, respectively. The occupancy of each protein was determined with quantitative PCR at the *RhoU* and *PLAU* gene promoter region encompassing the RBP-J binding site. ChIP using normal IgG was performed as a negative control. (**b**~**d**) Inactivation of Notch1 (DAPT) or JMJD3 (GSK-J4) or NF-κB (BAY 11–7082) results in decreased recruitment of NICD to the *RhoU* and *PLAU* gene promoters. Following DAPT, GSK-J4, or BAY11–7085 treatment, HaCaT keratinocytes were scratched and ChIP assay was performed.

Since our results demonstrated Notch1-dependent *RhoU* and *PLAU* gene activations, we next examined the recruitment of NICD to the genes in HaCaT keratinocytes after scratching by ChIP assay. Chromatin from HaCaT keratinocytes at 0~12 hr after scratching was immunoprecipitated with anti-NICD, and anti-acetylated histone H3, respectively. Quantitative PCR was performed to determine the occupancy of proteins using oligonucleotide primers encompassing RBP-J binding site (Supplementary Fig. S7b). Upon scratch wound, NICD significantly bound to the RBP-J binding site of *RhoU* and *PLAU* genes (Fig. 5a). We also detected an increased level of histone H3 acetylation (Fig. 5a). As expected, DAPT treatment resulted in a reduced level of NICD and acetylated H3 (Fig. 5b). In addition, the inactivation of JMJD3 and NF-κB also showed the same effects of DAPT on the level of NICD and H3 acetylation at the genes (Fig. 5c,d).

### Effect of JMJD3 and NF-κB on the Notch in skin wound healing

**Figure 6:**
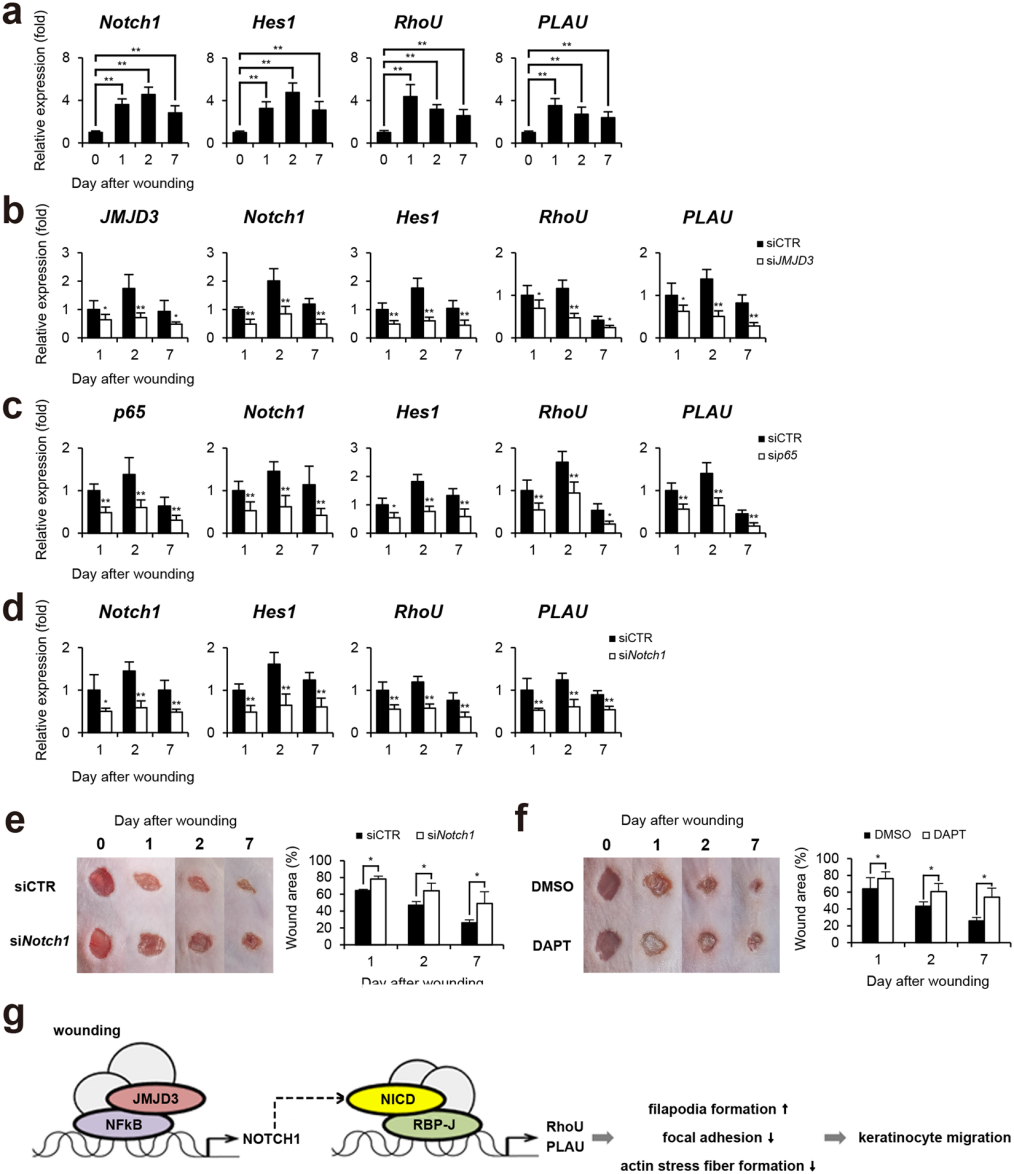
Delayed wound healing in Notch1-depleted mouse skin. (**a**) Transcripts of *Notch1*, *Hes1*, *RhoU*, *PLAU*, and *GAPDH* were determined in mouse skin wound by quantitative PCR (n = 6/group). (**b**~**d**) Following topical application of JMJD3 siRNA or NF-κB p65 siRNA or Notch1 siRNA in 30% Pluronic F-127 gel, wound tissue was harvested and transcripts of *Notch1*, *Hes1*, *RhoU*, *PLAU*, and *GAPDH* were determined by quantitative PCR (n = 6/group). (**e**,**f**) Depletion (siNotch1) or inactivation (DAPT) of Notch1 results in delayed skin wound closure. Following the application of Notch1 siRNA or DAPT, the wounded skins were photographed and a percentage of the wound area was measured quantitatively (n = 6/group). (**g**) A proposed model. Upon wounding, NF-κB p65 and JMJD3 binds to *Notch 1* gene promoter to activate *Notch 1* gene expression through demethylation of H3K27me3. The cleaved intracellular domain of Notch1 (NICD) binds to *RhoU* and *PLAU* gene promoters and activates *RhoU* and *PLAU* gene expression. These events result in enhanced filopodia formation, decreased focal adhesion and actin stress fiber formation in keratinocytes, leading to keratinocyte migration.

We further investigated the dependency of JMJD3 and NF-κB in Notch1-mediated wound healing using an *in vivo* mouse skin wound model. We first determined the expression of *Notch1*~*4*, *RhoU*, and *PLAU* in the skin after wounding. Quantitative PCR analysis revealed that the expression of the *Notch1* and *4* genes increased at 1~2 days after scratching and then decreased slowly (Figs 6a and S8a). Consistent to scratch-wounded HaCaT keratinocytes, *Notch1* is expressed more abundantly than *Notch4* in skin wounds (Supplementary Fig. S8b). The expression pattern of *RhoU* and *PLAU* genes is correlated with that of *Notch1* (Fig. 6a). Immunohistochemical results further showed that NICD is up-regulated and localized in the nucleus of wound edge keratinocytes, which are immunostained with anti-keratin14 (KRT14) antibody, at 1~7 days after wounding (Supplementary Fig. S9a).

To evaluate the necessity of JMJD3 and NF-κB for the up-regulation of *Notch1* expression in skin wound healing, JMJD3 or NF-κB p65 siRNA was applied topically to the skin wound. JMJD3 or p65 depletion attenuated the up-regulation of *Notch1*, *RhoU*, and *PLAU* expressions in wounded skin (Fig. 6b,c). We further found a decreased nuclear NICD expression in the wound edge after wounding (Supplementary Fig. S9b,c). Notch1 depletion also resulted in decreased *RhoU* and *PLAU* gene activations (Fig. 6d). Finally, we investigated the effects of Notch1 on skin wound healing processes. Although the wound was nearly closed by 7 days after wounding in a control siRNA or DMSO-applied skin wound, we found a significantly delayed wound closure in *Notch1* siRNA or DAPT-applied skin wound (Fig. 6e,f).

## Discussion

Notch pathway plays an important role in epidermal development in embryo and epidermal homeostasis in adult^8–11^. Notch family genes and its ligands are expressed in various epidermal layers during development^12–16^. Notch1 activates expression of keratin 1 and involucrin genes, which are early keratinocyte differentiation markers^44^. Deficiency of Notch1 further showed epidermal hyperplasia by reducing p21WAF/Cip1 expression in keratinocytes^44^. Notch signaling also regulates the proliferation and differentiation of keratinocyte stem cells located in basal layer of the epidermis^45^. However, a few reports have been reported the role of Notch signaling in keratinocyte wound healing.

In this study, we found that JMJD3 and NF-κB activate Notch1 expression at the wound edge in wounded keratinocytes. Although the expression pattern of Notch 4 was similar to that of JMJD3, Notch1 is expressed more abundantly than Notch4 in wounded keratinocytes and skin (Figs 1A and S1 and S6). Similarly, Notch1 has been considered as a primary Notch receptor in epidermal differentiation, proliferation, and inflammation^12, 15, 28–30^. Notch1 and its ligand Jagged1 are up-regulated at the wound edge in grafted human skin on the nude mouse^16^. Consistent to our findings, Notch1 is up-regulated in the epidermis including suprabasal and basal laminal keratinocytes at 1~4 days after wounding in the mouse skin^30, 46^. Up-regulated Notch1 activates TNFα expression, which can induce CCL20 and CXCL13 chemokine expression, leading to the recruitment of RORγ^+^ group3 innate lymphoid cells in wounded skin^30^. The inhibition of Notch by antisense RNA or γ-secretase inhibitor DAPT showed impaired skin wound healing due to inhibition of leukocyte infiltration, angiogenesis, and keratinocyte migration^47, 48^. DAPT-treated wound also showed incomplete wound closure^46, 49^.

Similar spatiotemporal expression of Notch1 and JMJD3 led us to investigate whether JMJD3 is required for Notch1 gene activation in wounded keratinocytes. In fact, depletion or inhibition of JMJD3 and NF-κB attenuated up-regulation of Notch1 gene expression. Consistently, JMJD3 and NF-κB were recruited to NF-κB binding sites on the Notch1 gene upon wounding as well as demethylation of H3K27me3. Our results are supported by requirement of NF-κB for the Notch1 gene activation^32, 50^.

At the wound edge, keratinocytes begin to rearrange their cytoplasmic and membrane structures, leading to disassembly of cell-extracellular matrix and cell-cell interaction for cell migration. These events include the actin cytoskeleton-mediated regulation of lamellipodia, filopodia, and focal adhesions during the early phase of wound healing^51, 52^. We found that depletion or inhibition of Notch1 results in decreased filopodia formation, increased focal adhesion and actin stress fibers, leading to delayed keratinocyte migration and wound closure. Our results further demonstrated that Notch1 directly up-regulates the expression of RhoU and PLAU gene, which are important regulators for cell migration (see below). Consistently, RhoU and PLAU have been identified as the target gene of Notch signaling^33, 35, 37^. RhoU is considered atypical Rho GTPase because it is likely to be predominately GTP-bound^53^. In fact, RhoU activates cell migration through formation of filopodia, loss of focal adhesion, and dissolution of stress fiber in several cell types^40–42, 53–56^. In particular, RhoU prominently localizes to focal adhesion, as indicated by vinculin expression^40^. Depletion of RhoU results in increased number of focal adhesion and decreased cell migration in response to wounding^40^. Filopodia formation by RhoU requires interaction with Pyk2 non-receptor tyrosine kinase in the presence of Src^41^. Pyk2 plays important role in cell adhesion and focal adhesion assembly through regulation of cytoskeleton dynamics^57–59^. PLAU converts plasminogen to plasmin, which cleaves ECM and MMPs for cell adhesion and migration^43^. Interestingly, PLAU is shown to be expressed in leading edge of migration keratinocytes^60–63^. In addition, PLAU colocalizes with vinculin at focal adhesion sites in various cell types^64–66^. Stimulation of uPA by Endo180, which is 180-kD transmembrane glycoprotein and complex with uPA and uPAR, leads to increased filopodia production in breast cancer cells^67^. Inhibition of uPA further decreases cell migration^68–72^. Interestingly, the expression and activity of MMPs are regulated transcriptionally and post-transcriptionally in wounded keratinocytes since we identified MMP-1, MMP-2, MMP-3, MMP-9, MMP-13, and MMP-14 as direct target of JMJD3 and NF-κB^23^ and PLAU-converted plasmin is shown to activate these MMPs^73–83^.

In conclusion, our study supports the important role of Notch1 as well as JMJD3 and NF-κB in keratinocytes wound healing. Specifically, JMJD3 and NF-κB-mediated Notch1 activation is required for the regulation of focal adhesion, filopodia, and stress fiber formation though RhoU and PLAU gene expression, leading to keratinocyte migration during the early phase of skin wound healing (Fig. 6g). Given that some genes related to Notch signaling are down-regulated in keratinocyte of venous ulcers and activation of Notch signaling in epidermal stem cells accelerate diabetic wound healing^84, 85^, our study may provide a new therapeutic intervention for the chronic skin wound.

## Materials and Methods

### Cell culture, animals, and chemicals

Human HaCaT keratinocytes were maintained in DMEM supplemented with 10% fetal bovine serum and antibiotics. Adult male ICR mice (8 weeks old; Samtako) were held in a temperature-controlled room (22 °C) at 55% humidity. The Committee for Experimental Animal Research at Sogang University approved the animal experiments and all methods were performed in accordance with guidelines and regulations relevant to the study. HaCaT keratinocytes were treated with 30 μM GSK-J4 (Tocris) and 10 μM BAY 11–7085 (Cayman) to inhibit JMJD3 and NF-κB, respectively. To inactivate Notch signaling, 10 μM DAPT (N-[N-(3,5-difluorophenacetyl)-l-alanyl]-S-phenylglycine t-butyl ester, Sigma-Aldrich) was used as a γ-secretase inhibitor.

### *In vivo* mouse skin wound healing

Mice were anesthetized by intraperitoneal injection of 300 μl of 1.25% Avertin (2,2,2-tribromoethanol, Sigma,-Aldrich), and full-thickness excision wounds using 4 mm biopsy punch (Kai) were made in the shaved dorsal skin. Wound tissue was harvested with 6 mm biopsy punch at the indicated time points (0, 1, 2, 7 days post wounding). After wounding, siRNA and DAPT were topically administered to the wound site. For the delivery of siRNA, 20 μl of 10 μM siRNA against mouse JMJD3 (m-063799-01-0005, Dharmacon), p65 (m-040776-01-0005, Dharmacon), Notch1 (18128, Bioneer) or control siRNA (d-001206-14-05, Dharmacon) mixed with 30% pluronic F127 gel (Sigma-Aldrich) was chilled on ice and applied to the wound cavity^86^. The efficiency of knock down of the specific gene was confirmed with real-time PCR. In addition, 20 μl of 100 μM DAPT was applied directly to the wound once daily until skin tissues were harvested^47, 49^. DMSO was applied as a control. The wounds were photographed and wound sizes were analyzed using ImageJ software (NIH, Bethesda, USA). Control and experimental wound were done on the same animal. Experiments were performed with two biological repeats (n = 3/group). Data were pooled (total n = 6/group).

### RNA interference

HaCaT keratinocytes were transfected with siRNA against human JMJD3 (m-023013-01-0005, Dharmacon), p65 (m-003533-02-0005, Dharmacon), Notch1 (4851, Bioneer), RhoU (58480, Bioneer), PLAU (5328, Bioneer), or control siRNA (d-001206-14-05, Dharmacon) using the X-treamGENE siRNA transfection reagent (Roche). The efficiency of knock down of the specific gene was confirmed with real-time PCR.

### RT-PCR

Total RNA was extracted from HaCaT keratinocytes and mouse skin using RNAiso Plus (Takara). First-strand cDNA synthesis from the total RNA template was performed with PrimeScript^TM^ RT master mix (Takara). The resulting cDNAs were subjected to real-time PCR using qPCR 2x PreMIX SYBR (Enzynomics) with a Stratagen Mx3000p (Agilent Technologies). PCR conditions used to amplify all genes were 10 min at 95 °C and 40 cycles of 95 °C for 15 s, 60 °C for 40 s. Expression data were calculated from the cycle threshold (Ct) value using the ΔCt method of quantification. *GAPDH* was used for normalization. Oligonucleotides used for real-time PCR are listed in Supplementary Table S1.

### Standard curve method for mRNA quantification

Plasmids containing each of Notch1 and 4 cDNA were used as standards for the absolute quantification. The concentration of each plasmid was measured by spectrophotometer or Ct value of real-time PCR using specific oligonucleotides for plasmid backbone. After concentration of plasmids were determined (same copies per 1 μl), plasmids were diluted (1:1, 1:10, 1:100, 1:1000, 1:10000). The diluted plasmids were amplified by specific oligonucleotides for each gene by real-time PCR. Using Ct value of each of plasmids, Notch1 and 4 standard curve were determined as according to the manufacturer’s instructions of Stratagene Mx3000 P (Agilent Technologies, Germany). The absolute amount of mRNA was calculated by each trendline equations [hNotch1: Y = −2.630*LOG(X) + 8.85, hNotch4: Y = −2.627*LOG(X) + 10.25; mNotch1: Y = −2.239*LOG(X) + 7.56, mNotch4: Y = −1.885*LOG(X) + 10.64].

### Over-expression of NICD

Intracellular domain of Notch1 was amplified using oligonucleotides: forward (EcoRI) 5′-CCGGAATTCGGTGCTGCTGTCCCGCAA-3′; reverse (XhoI) 5′-CCGCTCGAGCGTTTACTTGAAGGCCTCCGG-3′ and cloned into the pCS4 expression vector containing HA epitope. HaCaT keratinocytes were transfected transiently with jetPRIME reagent (Polyplus).

### Immunocytochemistry, immunohistochemistry, and histology

HaCaT keratinocytes were fixed for 15 min with 4% paraformaldehyde in PBS and permeabilized with PBST solution (0.5% Triton X-100 in PBS) for 30 min. After blocking of cells with 5% BSA in PBST solution for 1 h, cells were incubated with the anti-NICD (ab8925, Abcam) or anti-vinculin antibodies (V9131, Sigma-Aldrich) overnight at 4 °C. Antigens were detected with the secondary antibodies conjugated to TR or FITC (Sigma-Aldrich). To detect F-actin, cells were labelled with Alexa 546-phalloidin (Sigma-Aldrich). The wounded skins were immediately fixed with 4% paraformaldehyde in PBS and left overnight at 4 °C. The samples were dehydrated, embedded in paraffin and sectioned at 6 μm. The sections were de-paraffinized and boiled in Tris-EDTA buffer (pH 9.0) for 30 min, and then they were cooled at room temperature. The sections were incubated with 5% normal serum for 30 min. Subsequently, the sections were incubated with anti-NICD (ab8925, Abcam) or anti-KRT14 antibodies (ab7800, Abcam) overnight at 4 °C. After three washings with PBST, the sections were then incubated with a secondary antibody conjugated to FITC or TRITC. For histology, the sections were stained with hematoxylin and eosin (DAKO).

### Promoter reporter assay

HaCaT keratinocytes were transfected transiently with *Hes1* or *Notch1* gene luciferase construct in conjunction with a control thymidine kinase promoter-driven renilla luciferase. For mutation of RBP-J binding sites (TGTGGGAA → TGACGCTA and TTCACACG → TAGAGTCG) on *Hes1* gene promoter, PCR was performed^87^. After transfection of luciferase reporter containing gene and control renilla luciferase expression vector, cells were scratched and reporter activity was determined as described previously^88^.

### Western blot analysis

Cell extracts were prepared as described previously^89^. Western blotting was carried out using anti-NICD (4147, Cell Signaling) and anti-β-ACTIN antibodies (3G4-F9; AbFrontier), respectively.

### Scratch wound assay

HaCaT keratinocytes were plated on 6-well or 12-well culture plates and grown in 10% FBS containing a DMEM medium until they reached confluence. After cells were starved in the serum-free medium for 24 hr, the cell cultures were scratched using a sterile 10 μL tip. Cells were washed with serum-free medium and allowed to grow in 2% FBS containing DMEM. Cell cultures were recorded after scratching at the indicated times using the IncuCyte imaging system (Essen BioScience).

### Transwell assay

HaCaT keratinocyte migration was assessed quantitatively using Transwell (Corning Costar). After keratinocytes were starved in a serum free medium for 24 hr, keratinocytes (1 × 10^5^) were plated on the upper chamber of a 24-well Transwell plate with an 8μm pore size. After keratinocytes were incubated for 24 hr in a medium containing 10% FBS, the migrated keratinocytes were counted using the IncuCyte imaging system.

### Chromatin immunoprecipitation (ChIP)

ChIP was performed as described previously^89^. Anti-JMJD3 antibody (AP1022a, Abgent), anti-NICD (4147, Cell Signaling), anti-trimethyl H3K27 antibody (07–449, Millipore), anti-monomethyl H3K27 antibody (07–448, Millipore), anti-phosphorylated RNA polymerase II antibody (ab5131, Abcam), anti-p50 antibody (ab7971, Abcam), anti-p65 antibody (ab7970, Abcam), or normal IgG (SC-2027, Santa Cruz Biotechnology) were used. Real-time PCR was performed with a Stratagene Mx3000P using primers (see Supplementary Table S2).

### Statistical analyses

All quantitative data are presented as mean ± S.E.M. for three independent experiments. The differences between two groups were evaluated by a paired t-test. Analysis of variance (ANOVA) was used for multiple comparisons. Significance values were *P ≤ 0.05 and **P ≤ 0.01.

## Electronic supplementary material

Supplementary  information
