# Supplementary material for: JMJD3 and NF-κB-dependent activation of Notch1 gene is required for keratinocyte migration during skin wound healing
Source: Sci Rep. 2017 Jul 26;7:6494. doi: 10.1038/s41598-017-06750-7 (PMC5529578; doi:10.1038/s41598-017-06750-7)
Supplement: Supplementary file 1 — Supplementary information [file 41598_2017_6750_MOESM1_ESM.pdf]

Supplementary information

JMJD3 and NF-κB-dependent activation of Notch1 gene is required for keratinocyte migration during skin wound healing

Jungtae Na[1,3]*, Jee Yoon Shin[1]*, Hayan Jeong[1], Jee Youn Lee[2], Beom Joon Kim[3], Won Sun Kim[1], Tae Young Yune[2]**, and Bong-Gun Ju[1]**

[1]Department of Life Science, Sogang University, Seoul 04107, Korea

[2]Age-Related and Brain Diseases Research Center, School of Medicine, Kyung Hee University, Seoul 02447, Korea

[3]Department of Dermatology, College of Medicine, Chung-Ang University, Seoul 06973, Korea

Supplementary Figures

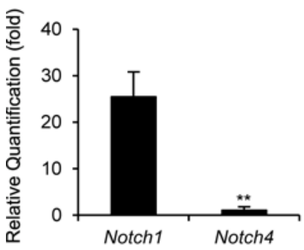

Figure S1. Expression level of Notch1 and 4 in scratch-wounded HaCaT keratinocytes. *Notch1* and *4* were quantified at 2 hour after scratching by standard curve method using real-time PCR. Plasmids containing each of Notch1 and 4 cDNA were used as standards for the absolute quantification, respectively.

.

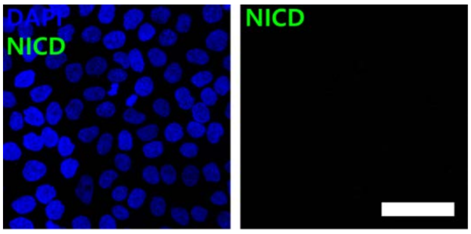

Figure S2. NICD expression in confluent monolayer HaCaT keratinocytes. Representative photomicrographs of NICD expression in HaCaT keratinocytes. Confluent HaCaT keratinocytes were immunostained with anti-NICD antibody. Nuclei were identified using DAPI staining. Scale bar, 25 μm.

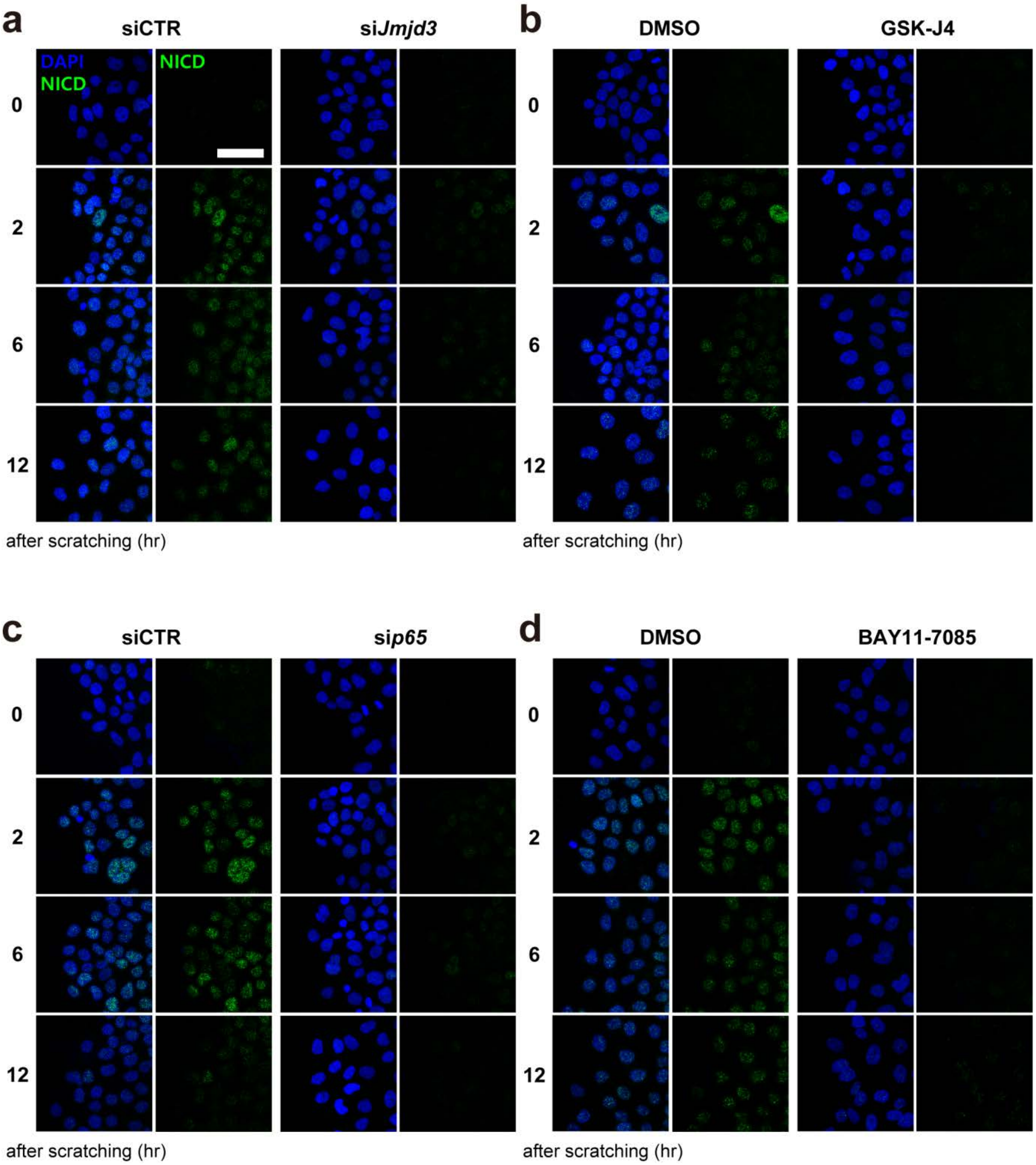

Figure S3. JMJD3 and NF-κB are required for the up-regulation of Notch1 expression in scratch-wounded keratinocytes. (a, b) Following the depletion (siJMJD3) or inactivation (GSK-J4) of JMJD3, HaCaT keratinocytes were immunostained with anti-NICD antibody. Nuclei were identified using DAPI staining. Scale bar, 25 μm. (c, d) Following the depletion (sip65) or inactivation (BAY11-7085) of NF-κB, HaCaT keratinocytes were immunostained with anti-NICD antibody. Nuclei were identified using DAPI staining. Scale bar, 25 μm.

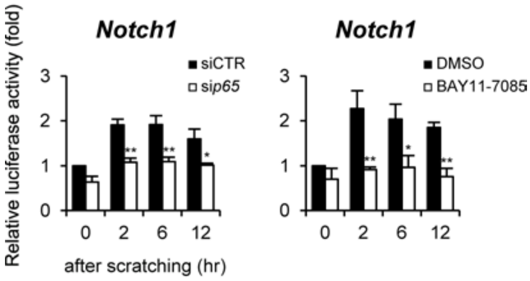

Figure S4. Suppression of Notch1 gene reporter activity by depletion or inactivation of NF-κB in scratch-wounded HaCaT keratinocytes. After transfection of *Notch1* gene-luciferase reporter vector and control renilla luciferase expression vector, HaCaT keratinocytes were scratched and reporter activity was determined.

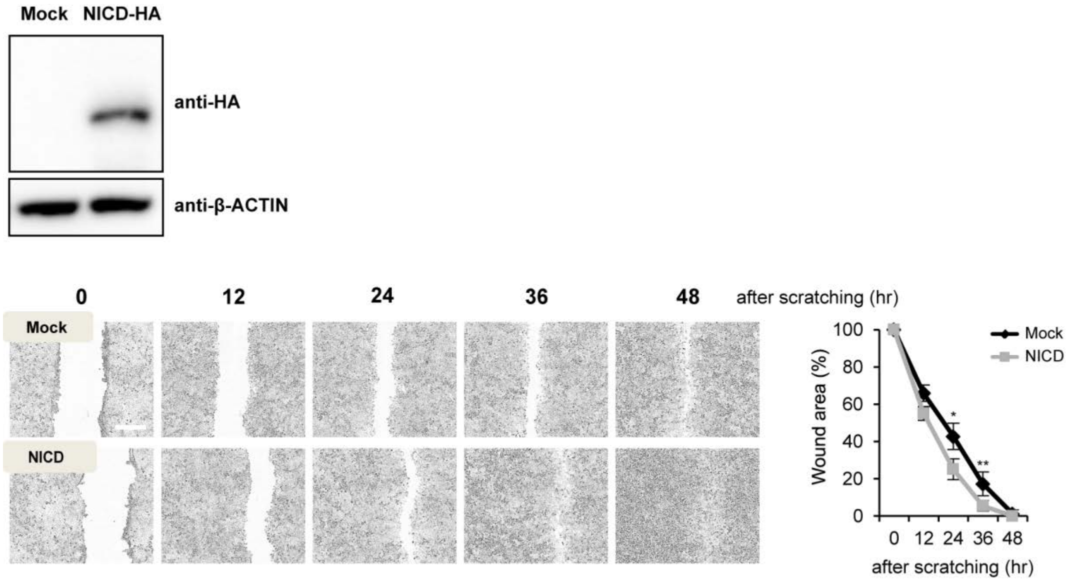

Figure S5. Over-expression of NICD accelerates wound closure in scratch-wounded HaCaT keratinocytes. After transfection of plasmids containing NICD cDNA in HaCaT keratinocytes, scratch assay was performed and photographed. Empty vector (mock) was used as negative control. Western blot analysis was performed to confirm the ectopic NICD expression using anti-HA antibody. Anti-β-ACTIN antibody was used as a loading control. Scale bar, 400 μm.

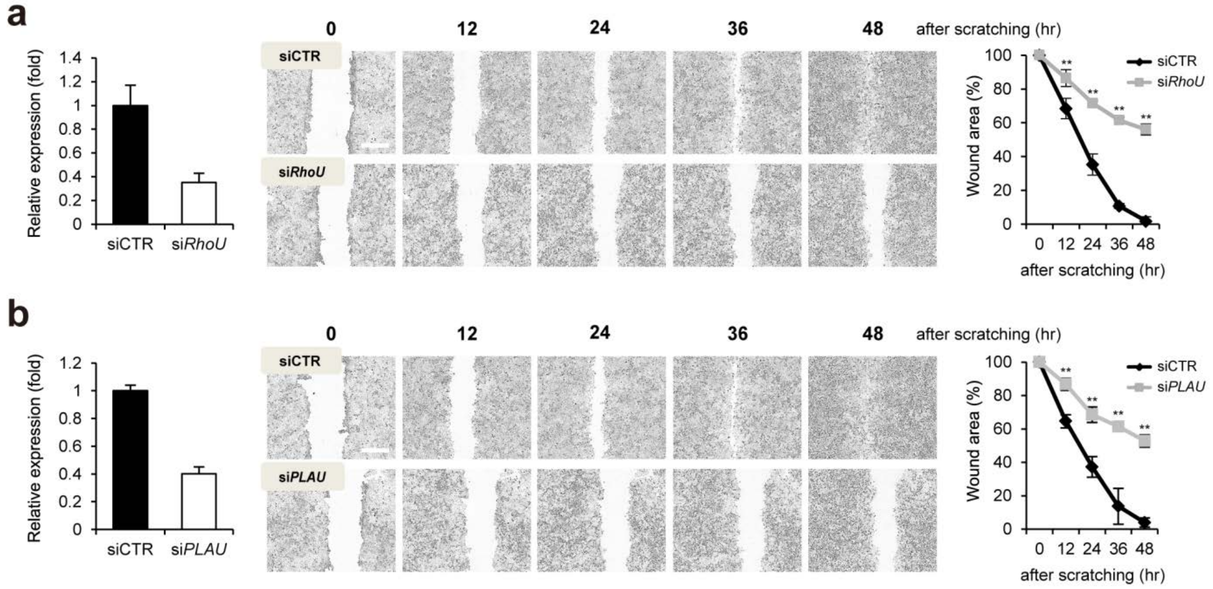

Figure S6. (a) Depletion of RhoU and (b) PLAU delay wound closure in scratch-wounded HaCaT keratinocytes. After transfection of siRNA against RhoU (siRhoU), PLAU (siPLAU), or control siRNA (siCTR) in HaCaT keratinocytes, scratch assay was performed and photographed. Scale bar, 400 μm. Expression level of RhoU, PLAU, and GAPDH were determined by quantitative PCR.

a **NF-κB binding sites in *Notch1* gene**

```
-2060   CTTCTCTGCTGGGATGGAGGCCCAAGGTGGAGGTGCTGTCGGCACAGCTGGGGCA
-2000   GACCAGGGGAGACCCCCTATCCAGGGATCCCCTCCTCCCCTCCCAGGCCGCCCCTCCGCA
                NF-κB                  NF-κB
-1940   AAATGCTGAGGACTGGCCCCCGGGCCTGGGCTACATGGGCCATTACTTCTGGCCTGGGGC
-1880   CCACAGGCAGAGATGCCAATGGAGGCACACAGGCAGTCGCACCCGCACCCGATCAGCACC
```

b **RBP-J binding site in *RhoU* gene**

```
-2436   TTCTTCTGTTTGTTTCTTTTTGAGACAGTGTCTCACTCTCGCCCAGGCTAGAGTATATTG
-2376   GTTGATCAAGGGCTCACTGGAGCCTCGACTTCCCAGGCTCAAGTGATCCTCCTACCTCAG
                                         RBP-J
-2316   CCTCTGGAGTATTTGGATTACAGGTGTGAGTCACCACACCTGGCTAATTTTTTTATTTTT
-2256   GGTAGAGACAGGGTTTCGCCATGTTGGCCAAGCTGGTCTCGAACTCGTGGCCTCAACCGG
```

RBP-J binding site in *PLAU* gene

```
-1234   GGAAACTTCCCAAAAGACCCGTTAACACTTCAATAGGAAGCACCAACAGTTTATGCCCTA
-1174   GGACTTTGTTCCCACAATCCTGTAACATCATATCACGACACCTAACCCAATCCTTATCAA
                  RBP-J
-1114   GCCCTGTCAAAAACGGACTTTAAACCAAGCTGCAAATTTTCAGTAATCTGGCCTTGCCTT
-1054   TCCCCCTCTGATAGCACCATCAAACAAACCCCCTTACTGCCGAAAGCAATAAGCCCGGCT
```

Figure S7. Nucleotide sequences in the upstream region of Notch1, RhoU, and PLAU genes. (a) NF-κB binding sites in human *Notch1* gene (b) RBP-J binding site in human *RhoU* and *PLAU* genes. Binding sites for NF-κB and RBP-J are underlined.

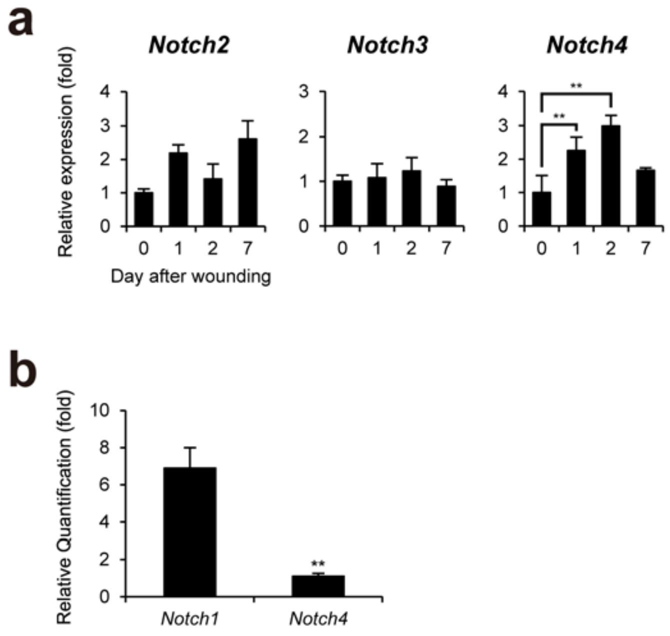

Figure S8. Expression level of Notch family genes in mouse skin wound. (a) Transcripts of *Notch2* ~*4*, and *GAPDH* were determined in mouse skin wound at 1 day after wounding by quantitative PCR. (b) *Notch1* and *4* were quantified by standard curve method using real-time PCR. Plasmids containing each of *Notch1* and *4* cDNA were used as standards for the absolute quantification, respectively.

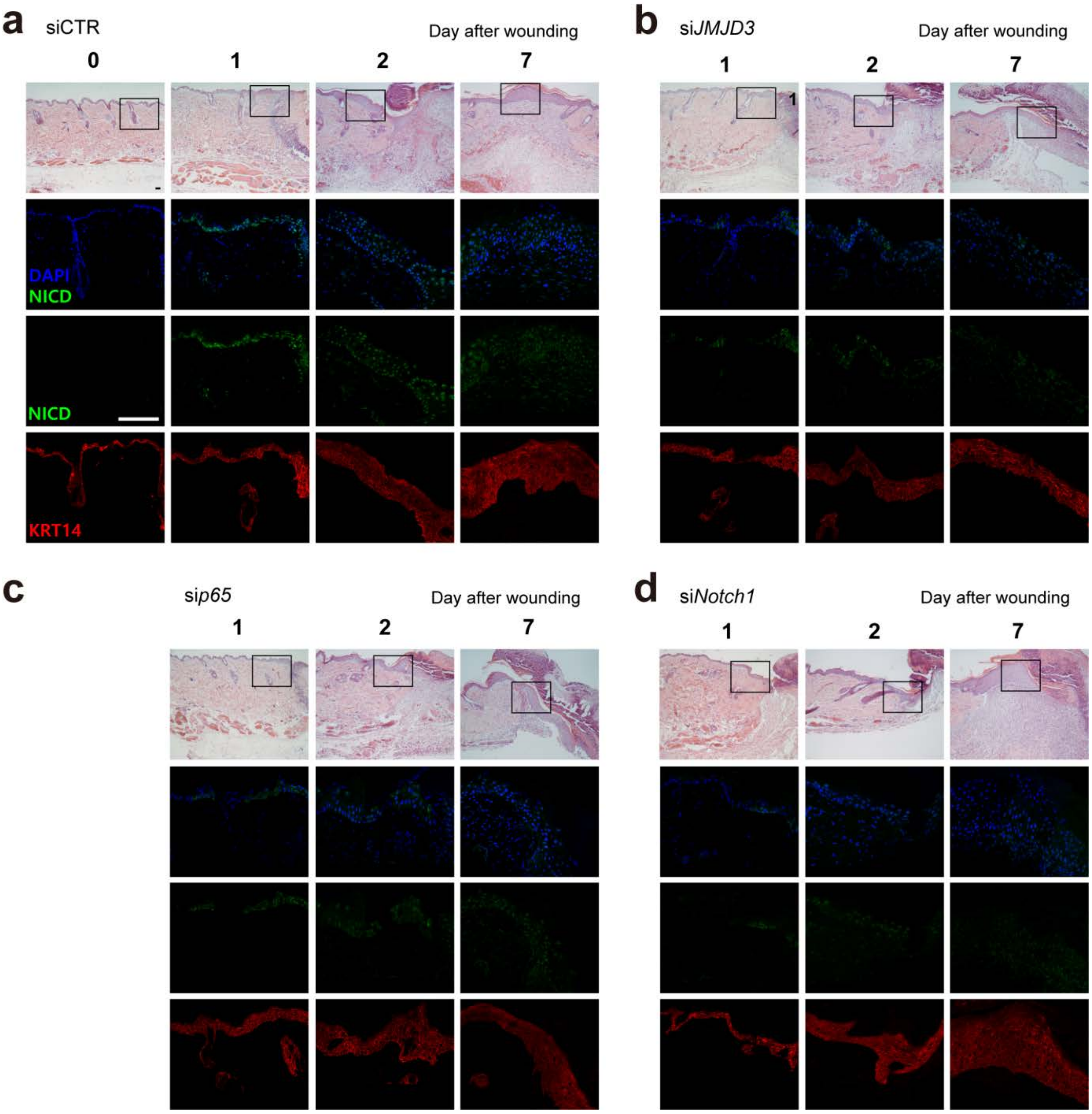

Figure S9. JMJD3 and NF-κB are required for up-regulation of Notch1 expression in wounded mouse skin. (a~d) Following the application of control siRNA, JMJD3 siRNA, NF-κB p65 siRNA, or Notch1 siRNA in 30% Pluronic F-127 gel, wound tissue was harvested at 1, 2, 7 day after wounding. Skin sections were immunostained with the anti-NICD antibody (green). To detect keratinocytes, sections were immunostained with the anti-KRT14 antibody (red). Nuclei were identified using DAPI staining. Scale bar, 100 μm.

Supplementary Tables

Supplementary Table S1. Oligonucleotide primers used for quantitative PCR in this study

Gene		human	mouse
**Gapdh**	Forward	CACCCACTCCTCCACCTTTGAC	AGGTCGGTGTGAACGGATTTG
	Reverse	GTCCACCACCCTGTTGCTGTAG	TGTAGACCATGTAGTTGAGGTCA
**JMJD3**	Forward	GCAGGGAAGAAAATCGCTTA	ACCCCTTCACGGGAAGTTG
	Reverse	ACAGCCCTCGCAGTGTCA	TCACTGTCGTGCTCTGATTCA
**p65**	Forward	ATGTGGAGATCATTGAGCAGC	AGGCTTCTGGGCCTTATGTG
	Reverse	CCTGGTCCTGTGTAGCCATT	TGCTTCTCTCGCCAGGAATAC
**Notch1**	Forward	GACCTCCCCAACACCTACAA	GATGGCCTCAATGGGTACAAG
	Reverse	TGCCGTTGTTAAAGCACTTG	TCGTTGTTGTTGATGTCACAGT
**Notch2**	Forward	GATCACCCGAATGGCTATGAAT	CTGTGAGCGGAATATCGACGA
	Reverse	GGGGTCACAGTTGTCAATGTT	ATAGCCTCCGTTTCGGTTGG
**Notch3**	Forward	GTCGTGGCTACACTGGACCT	AGTGCCGATCTGGTACAACTT
	Reverse	AATGTCCACCTCGCAATAGG	CACTACGGGGTTCTCACACA
**Notch4**	Forward	CACGTGAACCCATGTGAGTC	GAACGCGACATCAACGAGTG
	Reverse	TTGAGCAGTTCTGTCCATCG	GGAACCCAAGGTGTTATGGCA
**Hes1**	Forward	CCTGTCATCCCCGTCTACAC	GATAGCTCCCGGCATTCCAAG
	Reverse	CACATGGAGTCCGCCGTAA	GCGCGGTATTTCCCCAACA
**RhoU**	Forward	TCGTCGCTGGCATTCAATACT	GAGTGCTCAGCGTTGACTCAG
	Reverse	CCAGGACTTGGAGAGGTTTTTC	GACAGGTCCCGCACCTTATC
**PLAU**	Forward	GGGAATGGTCACTTTTACCGAG	ATGGAAATGGTGACTCTTACCGA
	Reverse	GGGCATGGTACGTTTGCTG	TGGGCATTGTAGGGTTTCTGA

Supplementary Table S2. Oligonucleotide primers used for ChIP assay in this study

Gene	Forward	Reverse
**Notch1**	TAGAGGATGGAGGCCCAAGGT	CTCAGCATTTTGCGGAGGG
**RhoU**	GCCCAGGCTAGAGTATATTGGTT	AAGCAGTCAGACCGGTTGAG
**PLAU**	CTTGCCTGCACAAATAAATGAA	ACAGGGCTTGATAAGGATTGG
